# Evaluation of CCL21 role in post-knee injury inflammation and early cartilage degeneration

**DOI:** 10.1371/journal.pone.0247913

**Published:** 2021-03-02

**Authors:** Mohan Subburaman, Bouchra Edderkaoui

**Affiliations:** 1 Musculoskeletal Disease Center, Research Service, VA Loma Linda Healthcare System, Loma Linda, California, United States of America; 2 Departments of Medicine, Loma Linda University, Loma Linda, California, United States of America; 3 Departments of Biochemistry, Loma Linda University, Loma Linda, California, United States of America; 4 Departments of Orthopedics, Loma Linda University, Loma Linda, California, United States of America; Universite de Nantes, FRANCE

## Abstract

The expression of some chemokines and chemokine receptors is induced during the development of post-traumatic osteoarthritis (PTOA), but their involvement in the pathogenesis of the disease is unclear. The goal of this study was to test whether CCL21 and CXCL13 play a role in PTOA development. For this purpose, we evaluated the expression profiles of the chemokines *Ccl21* and *Cxcl13*, matrix metalloproteinase enzymes *Mmp3* and *Mmp13*, and inflammatory cell markers in response to partial medial meniscectomy and destabilization (MMD). We then assessed the effect of local administration of CCL21 neutralizing antibody on PTOA development and post-knee injury inflammation. The mRNA expression of both *Ccl21* and *Cxcl13* was induced early post-surgery, but only *Ccl21* mRNA levels remained elevated 4 weeks post-surgery in rat MMD-operated knees compared to controls. This suggests that while both CXCL13 and CCL21 are involved in post-surgery inflammation, CCL21 is necessary for development of PTOA. A significant increase in the mRNA levels of *Cd4*, *Cd8* and *Cd20* was observed during the first 3 days post-surgery. Significantly, treatment with CCL21 antibody reduced post-surgical inflammation that was accompanied by a reduction in the expression of *Mmp3* and *Mmp13* and post-MMD cartilage degradation. Our findings are consistent with a role for CCL21 in mediating changes in early inflammation and subsequent cartilage degeneration in response to knee injury. Our results suggest that targeting CCL21 signaling pathways may yield new therapeutic approaches effective in delaying or preventing PTOA development following injury.

## Introduction

Osteoarthritis (OA) is a multifactorial disease characterized by progressive cartilage loss, bone remodeling, osteophyte formation and synovial inflammation with resultant joint pain that worsens over time. Osteoarthritis (OA) can be divided into two categories: primary and secondary. The cause of the primary OA is unknown and often occurs in elderly individuals. Secondary OA is due to either some medical conditions, or traumatic events, that affect individuals at any age leading to later development of OA. This specific type of secondary OA is termed Post-traumatic OA (PTOA). PTOA develops following injury due to unresolved inflammation, hemarthrosis, chronic destabilization of the joint and the biomechanical effects of the trauma on cartilage and other tissues. Acute innate inflammation that never completely resolves, occurs shortly after trauma, followed by anabolic responses, such as bone remodeling and extracellular matrix synthesis, which emphasizes the importance of inflammation post joint injury in PTOA development. PTOA has the particularity to affect both youth and elderly populations. Approximately 50% of individuals with anterior cruciate ligament (ACL) or menisci injuries develop PTOA within 10–20 years [1]. In United states, more than 5 million people suffer from PTOA [2] that represents a significant public health burden. As the cellular and molecular bases for the disease are not yet well understood, we were interested in determining whether we could identify new pathways that might serve as targets for the development of novel therapeutic interventions for PTOA to promote optimal long term health post joint injuries.

Recent studies reported elevated mRNA expression of chemokine *Ccl21* in the joints of both young and old mice in which injury was experimentally induced by medial meniscus destabilization (MMD) [3–5]. CCL21 was localized to chondrocytes, meniscal cells and the growth plate matrix. *Ccl21* levels were also increased in the MMD joints of young mice and in the old sham control joints. Furthermore, elevated levels of *Ccl21* expression were observed in synovial tissue from OA patients as compared to healthy patients [6]. CCL21 mediates its effects upon binding to chemokine receptor 7 (CCR7). It has been predicted that migration of T cells in response to inflammation is CCR7- and CCL21- dependent as CD4 (+) T cells use CCR7 to home to and migrate within lymphoid tissue where T-cell activation takes place [7]. CD4+ T cells are particularly important for the control of different immune responses during host defense and in the pathogenesis of different inflammatory diseases. Indeed, Zhu et al. [8], found the proportion of CD4+ T cell was significantly greater in OA patients compared to healthy patients. Inflammation in OA joints manifests with mononuclear cell infiltration observed in early and late stages of the disease. Accordingly, Hsieh et al. [9], reported the apparition of CD8+ cells at the synovial membrane 30 days post anterior cruciate ligament-transection. These data suggest that inflammatory cells arrive at the synovia in response to knee injury and chronic abnormal loading.

Based on the above findings and the established role of CCL21 in mediating T cell responses to inflammation, we hypothesized that CCL21 might also be a critical contributor to PTOA development. To test this hypothesis, we chose an established rodent model of surgically induced injury, partial medial meniscectomy and destabilization (MMD). Rodents undergoing MMD display degenerative changes typical of PTOA within 3–4 weeks after surgery [10]. We evaluated the expression pattern of *Ccl21* during development of PTOA post-MMD. Since *Ccl21* expression levels appear to precede the development of PTOA, we then examined whether inhibition of CCL21 function using CCL21 antibody will be effective in limiting or preventing development of the disease.

## Materials and methods

### Surgical procedures

Surgeries were performed on 10-12-week-old male Sprague-Dawley rats (Charles River Laboratories, Hollister, CA, USA), and on Balb/c mice (The Jackson Laboratory, Bar Harbor, ME, USA). Balb/c mice were only used to confirm the response of *Ccl21* mRNA expression to MMD over an extended time during the development of PTOA. All procedures performed on animals were approved by the Institutional Animal Care and Use Committee at the VA Loma Linda Health Care System, USA (ACORP# 0115-01-15 and 0004-01-12), and by the USAMRMC Animal Care and Use Review Office (ACURO). Under general anesthesia with isoflurane, the medial side of each knee joint was shaved and the skin around the incision area cleaned with 70% ethyl alcohol.

Knee joint instability was induced surgically by partial medial meniscectomy (MMD) as previously described [10, 11]. Briefly, the right knee (Rk) was opened with an incision from the distal patellar tendon to the proximal tibia plate, and a perpendicular incision from the patella to the medial collateral ligament (MCL). The MCL was transected below its attachment with the meniscus. The patellar ligament was pulled out to visualize the meniscus, and the medial meniscus grasped with a hemostat and transected from two sides, taking care not to damage the tibial surface. The left knee (Lk) was not operated or was sham operated through the same approach but without any ligament or meniscus transection and served as control. Following surgery, tissue debris was removed by rinsing with sterile saline, and the incision closed in two layers. The joint capsule and skin were sutured as previously described [11]. After surgery, animals received one intra-peritoneal injection of Buprenorphine at 60 μg/kg to alleviate post-surgery pain. Animals were sacrificed at different time points post-surgery (S1 Table). Animals were euthanized, in their own home cages, by carbon dioxide inhalation for 3–5 min from a compressed CO_2_ gas tank, followed by cervical decapitation. Then, the samples were collected for processing to determine expression levels and/or for histological preparations (S1 Table).

### Treatment with anti-CCL21 antibodies

To block the function of CCL21, at one day post-surgery animals were put under general anesthesia with isoflurane. 10 μg of rabbit anti-rat polyclonal CCL21 antibody (cat# ab25119, Abcam Inc., Cambridge, MA, USA), diluted in 20 μl Phosphate-buffered saline (PBS), was injected in rat MMD-operated knees under the patella ligament between femur and tibia plate, using insulin syringe and 31G needle inserted into the joint space towards medial side. The knees of control rats were injected with 20 μl PBS.

### Gene expression quantification

Animals were sacrificed at different time points post-surgery (S1 Table). Since we expected infiltration of inflammatory cells to the synovial space very early post-surgery, we collected synovial tissue separated from the other tissues at one day post-surgery; anterior synovial membrane and fat pad were harvested together from both MMD-operated knees and control-sham-operated knees for the RNA quantification of inflammatory cell markers (S1 Table). Whole knee was also collected by excising a region of the knee joint between the tibia and femur growth plates for RNA extraction and quantification of mRNA levels of genes of interest (S1 Table). Tissues collected for RNA extraction were snap frozen in liquid nitrogen, and stored at -80°C.

#### Real-time quantitative polymerase chain reaction

Samples were pulverized in liquid nitrogen; total RNA was isolated using Trizol and RNeasy kit (Qiagen, Redwood City, CA, USA) and processed for reverse transcription (RT)-polymerase chain reaction (PCR). Then, Real-time quantitative PCR (qPCR) was performed using pre-designed primers (S2 Table), the Applied Biosystems ViiA7 RT-PCR systems instrument, and the SYBR Green PCR kit from Applied Biosystems Inc., as previously described [12]. Synovial tissue was used to evaluate the expression of three T cell markers, *Cd3*, *Cd4* and *Cd8* at one day-post-surgery only (S1 Table). The whole knee was used to evaluate the expression of inflammatory cell markers as well as other inflammatory effectors, such as the cytokines *Il-6*, *Tnfa*, and chemokines *Ccl21* and *Cxcl13*, and the expression of matrix metalloproteinases, *Mmp3* and *Mmp13* at various time points (S1 Table).

### Histology assessment of knee joints

MMD, sham and un-operated knee joints were collected from rats after euthanasia and muscle tissue removed. Knees were fixed for three days in 10% buffered formalin, decalcified in either EDTA for 30–34 days, or in Formical4 decalcifier (cat# 1214–1, StatLab, McKinney, TX, USA) for 6 days, and then embedded in paraffin. Starting from the posterior/anterior side of the joint, 5 μm sections were taken at 100-μm intervals. Slides were stained with hematoxylin-eosin (H&E) to assess inflammation early post-MMD, and with Safranin-O/Fast green to assess general morphology and matrix proteoglycans. Samples were independently evaluated by two observers, to prevent bias.

To assess early post-surgery inflammation, both MMD- and sham- operated knees were used. Three H&E stained sections from approximately same knee joint areas were evaluated from each rat to compare cellular density at the synovium. Stained sections from knee joints collected at days 1 and 3 post-MMD were examined by light microscopy, and the number of nuclei were counted from the images taken using 20x microscope lenses. Data was quantitated using ImagePro.Plus software.

Microscopic examination of the articular cartilage was graded using Mankin’s histology grading system [13]. This scoring system assesses the changes in structure (0–6 points), cellularity (0–3 points), matrix staining (0–4 points), and tidemark integrity (0–1 points), for a maximum of 14 points. The final score for each cartilage was based on the most severe histologic changes observed in 4–6 histology sections from each specimen. Sections were selected in approximately the same areas for all animals. Cartilage damage was evaluated on medial side that was operated from each knee because collateral side was not significantly affected by the surgery.

### Immunohistochemistry staining to assess secretion of CCL21 and the infiltration of inflammatory cells in response to MMD

Immunohistochemical staining, for IL-6, CCL21 and for CD4+ and CD8+ cells, was performed in dewaxed paraffin sections. Sections were dewaxed in Histochoice (VWR, Visalia, CA, USA), passed through graded alcohols, and rehydrated with water. Then, antigen retrieval was performed by incubating sections in bowled citrate buffer for 15 min at room temperature. After blocking with Protein Block (Abcam kit, Burlingame, CA, USA), sections were incubated overnight with rabbit anti-rat polyclonal IL-6, CD4-antibody (Novus Biological, CO, USA), CCL21-antibody (Invitrogen/Thermo Fisher, Scientific, Waltham, MA, USA) or mouse anti-rat monoclonal CD8 antibody (Thermo Fisher) at 4°C. Then, the sections were treated with hydrogen peroxide blocking reagent at room temperature and were processed using EXPOSE Mouse and Rabbit specific HRP/DAB Detection IHC Kit (Abcam kit, cat#80436) according to the manufacturers’ instructions, followed by counterstaining with Mayer’s hematoxylin solution (IHC World LLC, Ellicott city, MD, USA). As negative control, 2.5% normal goat serum was used to confirm specific staining of secondary antibodies. Stained sections were examined by light microscopy. Data was quantitated using ImagePro.Plus software. For quantification, 3 sections at 100 μm interval were analyzed from each knee, and for CD4 and CD8, we focused on synovial tissue at the joint capsule.

### Statistical analysis

The mean and standard error (SE) were used to illustrate the results from 4–8 replicates for each experiment. Statistical significance (P≤ 0.05) was assessed using one-way ANOVA with post hoc Tukey honest significant difference (HSD).

## Results

### Histology assessment of knee joints after MMD

**Fig 1:**
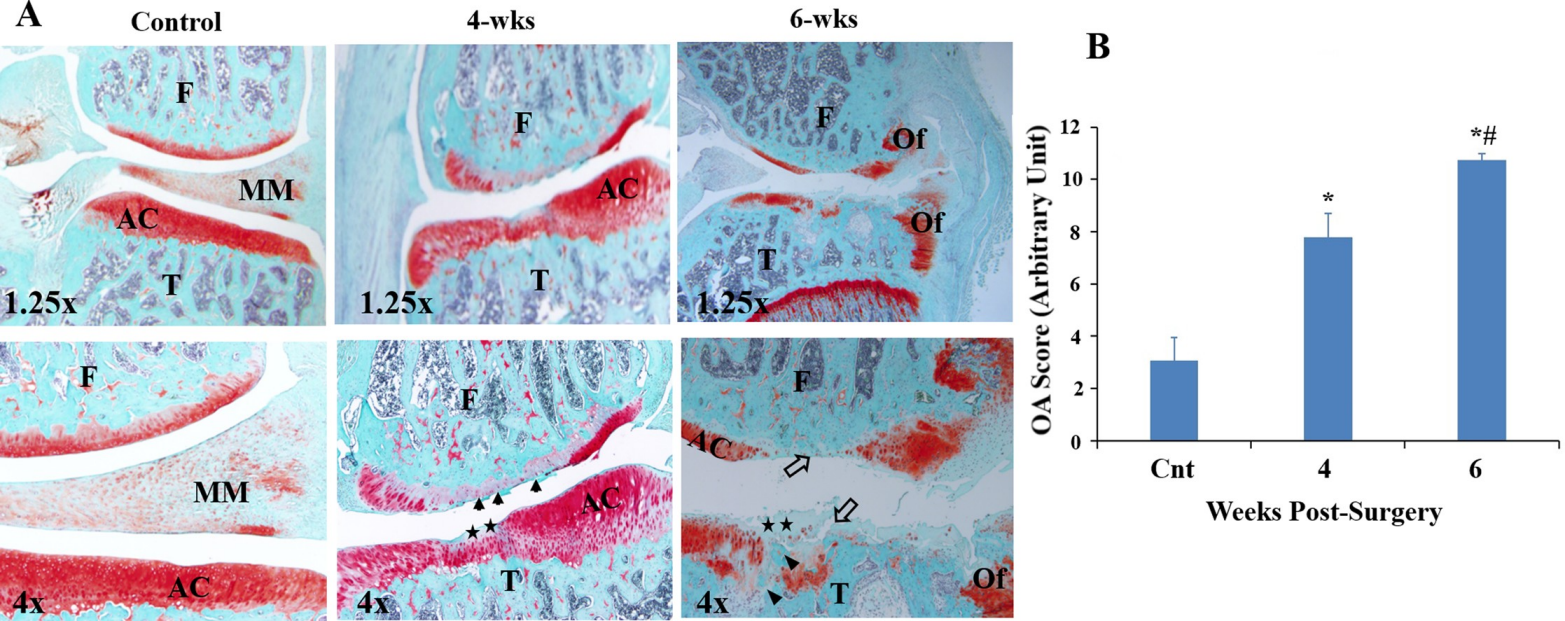
Histopathological evaluation of cartilage of rat knee joints post-MMD at 4 and 6 weeks post surgeries. **(A)** Representative images of histology sections from the medial side of knee joints isolated from, a control unoperated 17-week-old rat, a 15-week old rat at 4 weeks post-surgery (4-wks), and 17-week-old rat, at 6 weeks post-surgery (6-wks). The section from control unoperated knee shows smooth with no disruption of the articular cartilage surface integrity. Representative image of the medial condyle and tibia plateau of the MMD-knee, isolated at 4 weeks post-surgery, shows irregularities at the superficial layer of the articular cartilage (AC), fibrillation (black arrows) and loss of Safranin-O staining at the superficial zone (black stars). Image of the medial condyle and tibia plateau, isolated at 6 weeks post-surgery, shows severe cartilage degeneration, loss of Safranin-O staining (black stars) and depletion of chondrocytes (open arrows) with irregular trabecular bony between the junction of calcified cartilage (black arrowhead), and osteophyte formation (Of). Sections were stained with Safranin-O/Fast Green and observed using microscope objective lenses 1.25x and 4x., AC. for articular cartilage; Of. for osteophytes; T. for tibia plate and F. for femoral condyle. **(B)** Microscopic examination of the safranin-O stained sections. The changes in the articular cartilage were graded based on the changes in structure (0–6 points), cellularity (0–3 points), matrix staining (0–4 points), and tidemark integrity (0–1 points), and has a maximum of 14 points. The final score for each knee joint was based on the most severe histologic changes observed from multiple sections of each specimen. Data are presented as mean of 6 knees ± SE, n = 6. *P<0.05 *vs* control unoperated knees, #P<0.05 *vs* MMD-Knees at 4 wk-post-surgery.

To evaluate the time course of pathological effects caused by MMD-induced injury on the articular cartilage degeneration and PTOA development, rats were sacrificed at two time points, and knees from MMD-operated as well as control un-operated knees were collected and processed for histological analysis. As expected, the superficial layer of articular cartilage was smooth, cellularity was not affected, and no significant disruption of surface integrity was observed in unoperated control knees (Fig 1). Safranin-O staining was preserved, and a smooth bony trabeculum was observed in the subchondral layer (Fig 1). In contrast, 4- weeks post-surgery, MMD-operated knees showed irregularities and fibrillation in the superficial layer of the articular cartilage, and loss of proteoglycan as evidenced by reduced Safranin-O staining (Fig 1). At 6 weeks post-surgery, MMD-operated knees showed sever loss of Safranin-O staining, not only at the superficial zone but also in the calcified cartilage layer (Fig 1). We also observed fibrillation and depletion of chondrocytes in the cartilage layer, as well as cleft and irregular bony trabeculum between the junction of calcific cartilage and subchondral layers (Fig 1). Osteophyte formation was indicated by a fibrocartilage capped bony outgrowth lining the side of condyle and tibial plate (Fig 1). In addition, significant differences in Mankin scores were observed between the control un-operated knees and MMD-knees, and between the MMD-knees at 4 and 6 weeks post-injury (Controls = 3.06 ± 0.88; 4 weeks post MMD = 7.80 ± 0.9, *P*<0.05; 6 weeks post-MMD = 10.75 ± 0.22, P<0.05) (Fig 1B).

**Fig 2:**
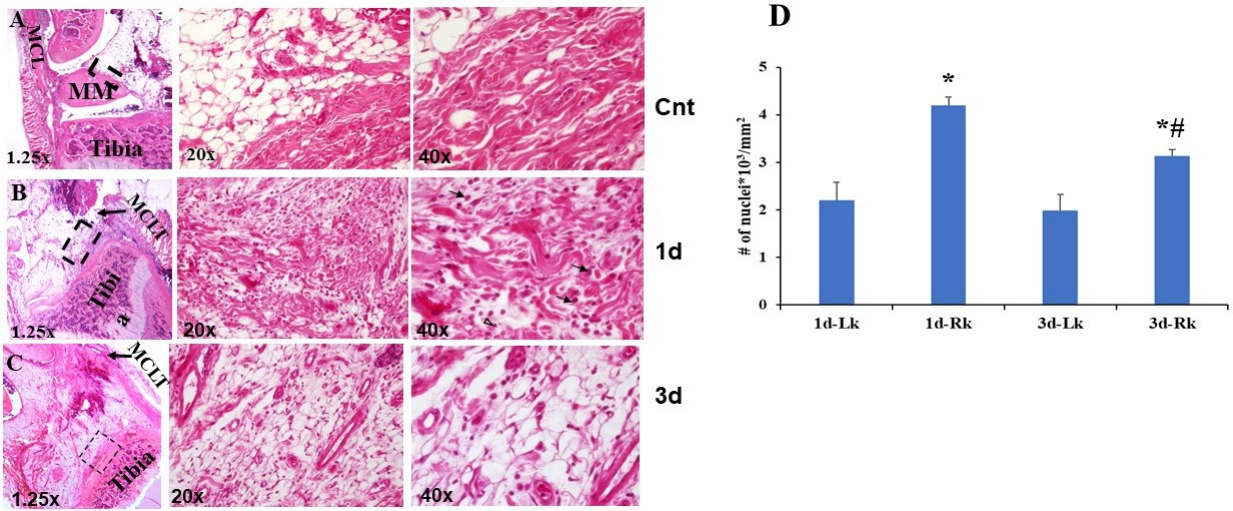
MMD caused recruitment of inflammatory cells to rat injured knees as early as one day post-MMD. Hematoxylin-Eosin (H&E) staining of histology sections collected from sham-operated left knee joint **(A)**, MMD-operated knee joint collected, at one day post-surgery **(B)** and 3 days post-surgery **(C).** Dashed black squares in the left panels indicate the regions magnified at the right panels. Inflammatory cells are obvious in the synovium of MMD-knee joint with abundant granulocytes (black arrows) and mononuclear cells (open arrowheads) at one day post-surgery. **D.** Cell density quantification at one and three days post-MMD in the synovial tissue of rat knee joints. Nuclei were counted in 2 fields per histology section, close to articular cartilage. 3 histology sections were evaluated from each knee. The sections were chosen in approximately the same knee joint area for comparison. We have counted the number of nuclei per mm^2^ synovial space in the MMD-operated right (Rk) knees and contralateral left knee (Lk) joints. Data are presented as mean ± SE, n = 6 right knees (Rk), *P<0.05 vs Lk sham-operated, #P<0.05 between Rks of 1d- and 3d- post surgery.

To assess early inflammatory response to MMD, we have used MMD- and sham-operated knees. A brief histologic assessment of synovial tissue at days 1 and 3 post-surgeries was performed. At one day post-surgery, analysis of knee sections stained with Hematoxylin-Eosin (H&E) revealed hyperplasia around the joints (Fig 2B). More cells were present in the synovium of MMD-operated knees (Fig 2B and 2C) compared to control sham-operated knees (Fig 2A) processed at the same time. We have noticed some granulocytes and mononuclear cells in MMD-knees (Fig 2B at 40x). Cell density increased significantly at the synovial space, both at one- and three- days post-surgery in response to MMD as evidenced by the number of nuclei per mm^2^ (Fig 2D) in MMD-operated knees compared to sham operated knees. As expected, at three days post-surgery (Fig 2C), inflammation was reduced as evidenced by the presence of less inflammatory cells at the synovium of operated knees, compared to one day post-surgery (Fig 2D). but cell density remained greater in MMD-knees compared to sham operated knees.

### Gene expression changes following MMD

**Fig 3:**
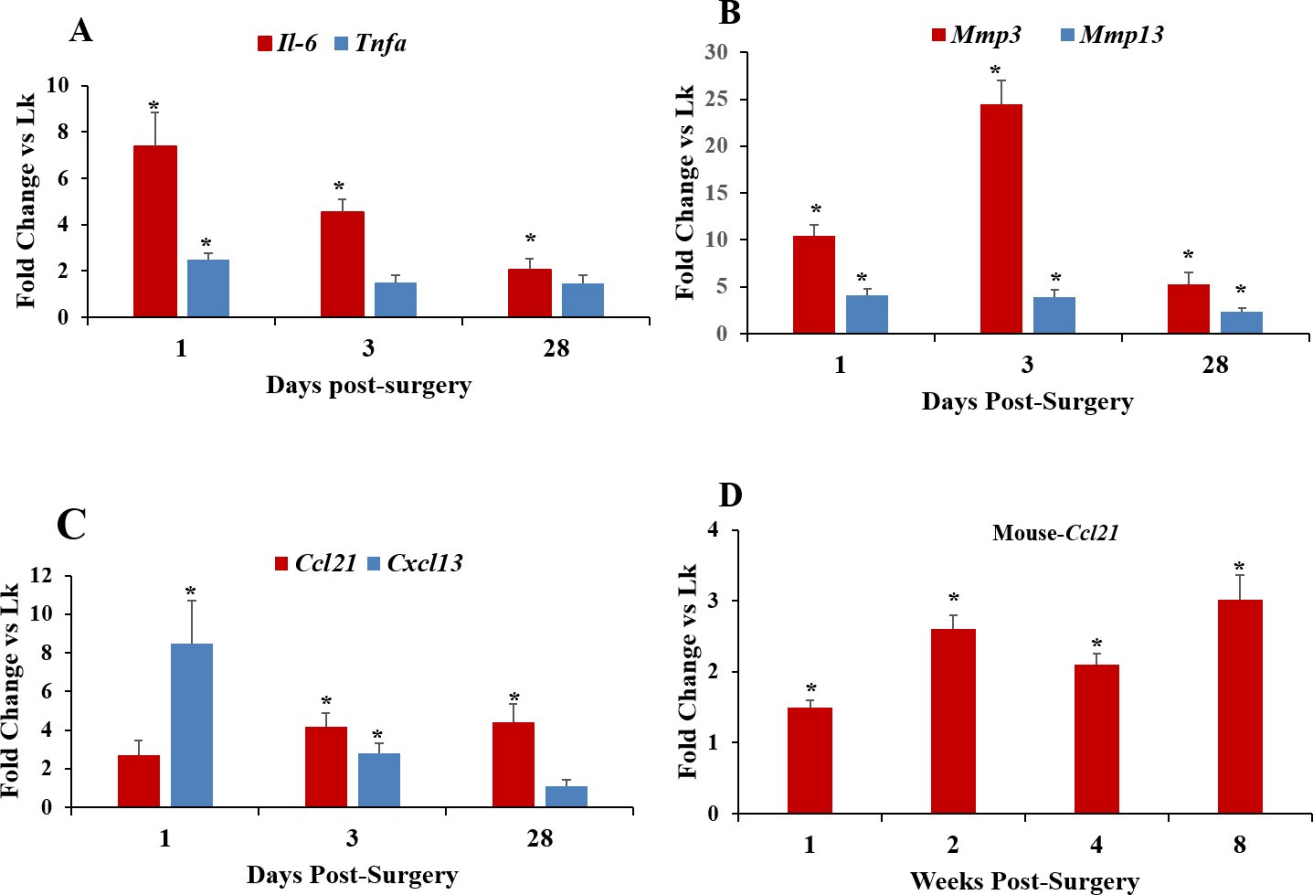
Evaluation of the changes in mRNA expression levels of different genes in rodent knee joints at different time points post-MMD. Two inflammatory cytokines, *Il-6* and *Tnfa* **(A)** and two major matrix metalloproteinases, *Mmp3* and *Mmp13* in rat knees **(B);** The two chemokines of interest in this study in rat knees **(C)** and *Ccl21* in mouse knees **(D).** Control unoperated knees and MMD-operated knees were collected from rats at three time points post-surgeries (**A, B, C**), and from mice at 4 time points (**D**). RNA was isolated from both MMD-operated and control unoperated knees, and mRNA expression level was evaluated by qPCR using pre-designed primers. Data are expressed as fold change in response to MMD as compared to control unoperated knees (Lk), and are presented as mean ± SE, n = 6 and *P<0.05 *vs* control un-operated knees.

We measured mRNA levels, in both MMD- and control un-operated rat knees, for two cytokine markers of inflammation, *IL-6* and *Tnfa*. At one day post-MMD, *Il-6* mRNA levels were 7-fold higher than controls, while *Tnfa* levels were 2-fold higher (Fig 3A). By three days, *Tnfa* mRNA levels had returned to normal and remained that way at 28 days. In contrast, while *Il-6* mRNA levels also declined at 3 and 28 days, they remained elevated compared to controls at all time points examined (Fig 3A).

We also measured mRNA levels of two matrix metalloproteinases involved in extracellular matrix degradation, *Mmp3* and *13*. The results showed that following MMD, *Mmp3* mRNA levels in rat operated knees were increased by 10-fold within one day, 24-fold at 3 days, and 5-fold at 28 days. *Mmp13* mRNA levels also increased, but to a lesser extent being 4-fold greater at 1 and 3 days, and 2-fold greater than controls at 28 days (Fig 3B). Expression levels of *Ccl21* and *Cxcl13*, two chemokines known to exert chemotactic effects on different T cell subsets, were also measured in rat knees to determine whether they were affected by MMD. Our results showed that *Ccl21* mRNA levels were slightly but not significantly increased in response to MMD at 1-day post-surgery (Fig 3C). However, at 3- and 28-days post-MMD, *Ccl21* mRNA levels were significantly increased compared to controls (Fig 3C). *Cxcl13* mRNA level was found to be elevated as early as 1- day post-MMD, remained elevated at 3- days post-MMD, but returned to normal levels by 28-days post-MMD (Fig 3C). These data show that expression of both *Cxcl13* and *Ccl21* increases as part of the post-injury inflammatory process, but since only *Ccl21* mRNA levels remain elevated, it is possible that *Ccl21* expression plays a major role in the development of PTOA.

We, further, examined *Ccl21* mRNA expression levels during PTOA development in mouse MMD-knee joints, over an extended time course, to confirm the involvement of CCL21 in PTOA development post-MMD. Our results showed that *Ccl21* mRNA levels were elevated for at least 8 weeks following surgically induced injury in mice (Fig 3D).

**Fig 4:**
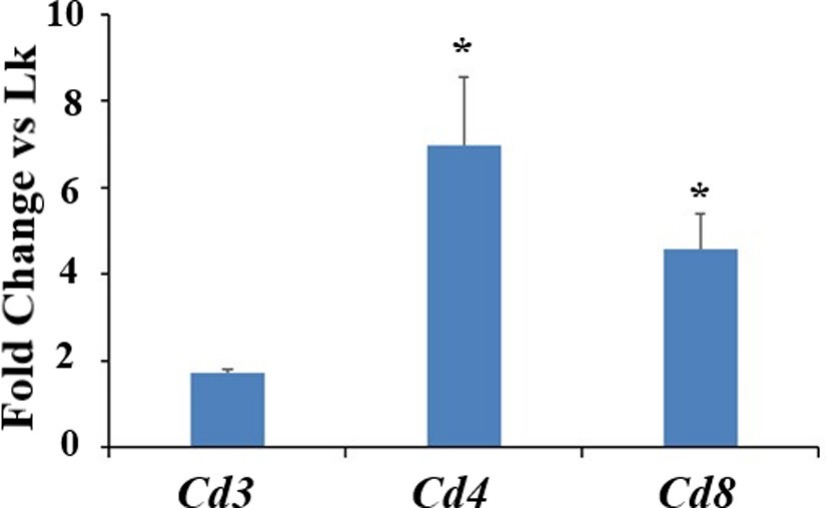
MMD caused the recruitment of CD4 and CD8 positive cells to rat injured knees at one day post-MMD. Quantification of mRNA expression levels for the markers of three T cell subsets isolated from rat knee joint synovial tissue, at one day post-surgery. Both right MMD-operated knees and left sham-operated knees (Lk) were collected. Data are expressed as fold change *vs* control sham-operated knees and are presented as mean ± SE, n = 4–5, *P<0.05 *vs* control sham-operated knees (Lk).

To determine if the increased expression of *Il-6* and the chemokines of interest early post-surgery is related with a raise in inflammatory cell recruitment, we examined mRNA levels of markers for T cell subsets (*Cd3*, *Cd4* and *Cd8*) in samples of rat synovial tissue isolated at day one post-surgery. mRNA levels of *Cd4* and *Cd8* in synovial tissue were increased by 7- and 4.6-fold respectively, in MMD-knees relative to control sham-operated knees (Fig 4). *Cd3* expression was slightly, but not significantly increased (+1.7-fold, P = 0.09) in MMD knees (Fig 4).

#### Il-6 and CCL21 expression in MMD-knee joints-immunostaining

**Fig 5:**
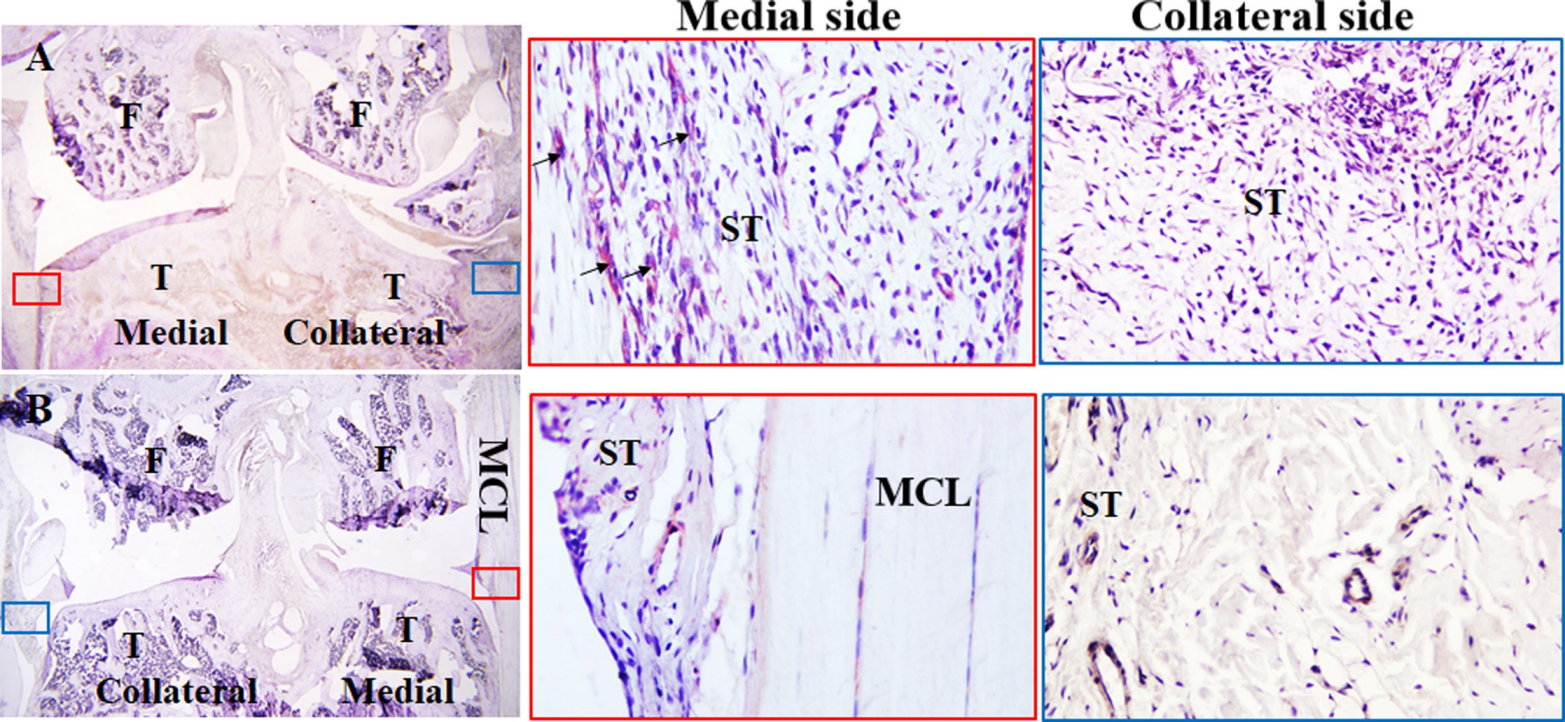
IL-6 synthesis is induced in response to MMD at three days post-surgery. Immunohistochemistry staining was performed using antibodies against rat IL-6 for both rat MMD- (**A**) and the left sham-operated knees (**B**) collected at day 3 post-surgery. Representative images of histology sections collected from rat MMD- (**A**) and sham-operated knees (B), brown color represents IL-6 positive areas. Arrows show some of IL-6 positive stained areas. The squares in the left panels represent the areas magnified 20x on the right panels. T. tibia plateau, F. femur condyles, ST, synovial tissue. MCL, medial collateral ligament. **A** and **B** were taken using 1.25x microscope lenses.

**Fig 6:**
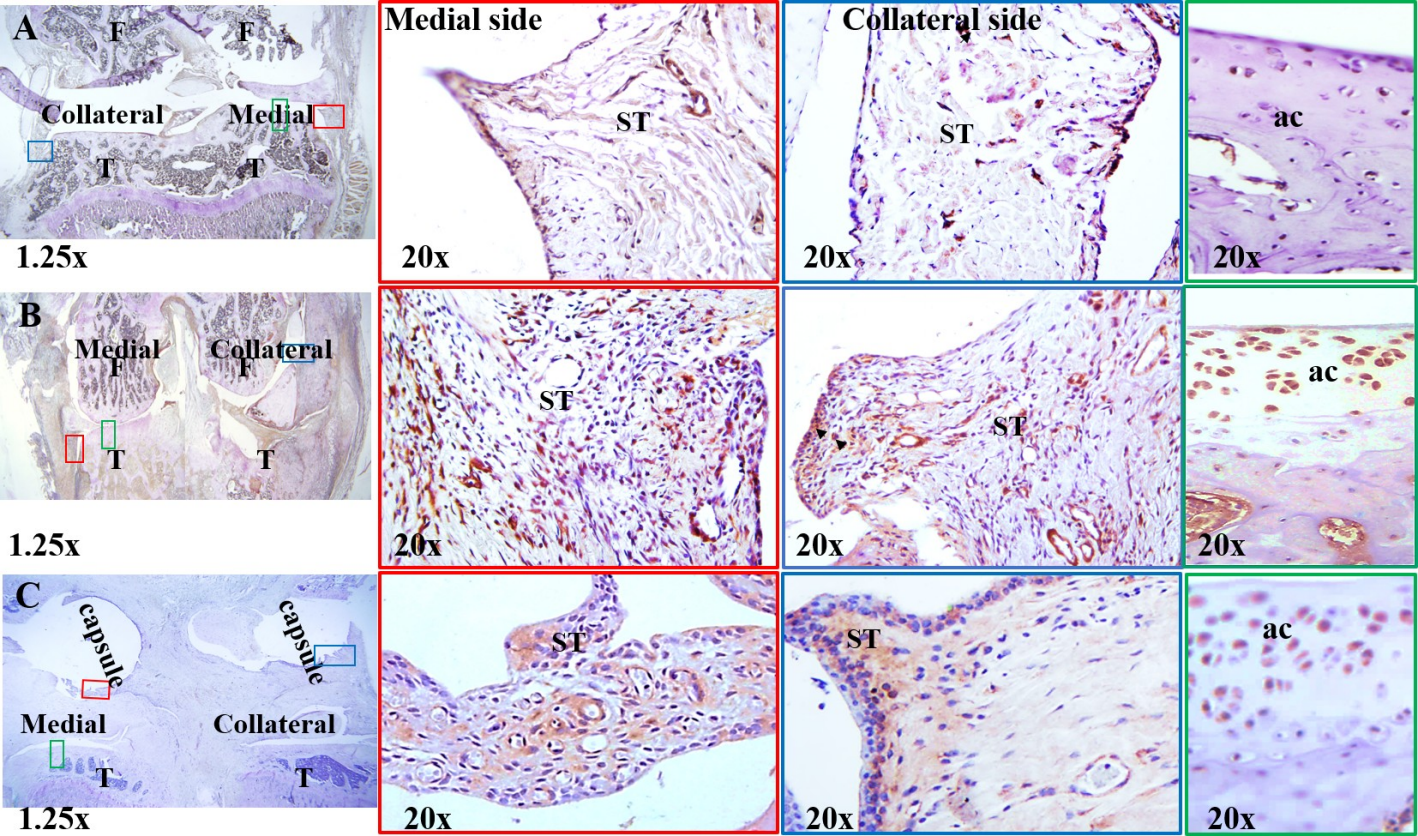
CCL21 synthesis was increased in response to MMD compared sham-operated knees. Immunohistochemistry staining was performed using antibodies against rat CCL21 antibodies for both sham- (**A**) and MMD- operated knees (**B** and **C**), collected from rats at day 3 (**A** and **B**) and 4 weeks (**C**) post-surgeries. Representative images of histology sections collected from Sham- (**A**) and MMD-operated knees (**B and C**), brown color represents CCL21 positive areas. Arrowheads show some of CCL21 positive stained areas. The small squares on the left panels represent the areas magnified 20x on the right panels. T. tibia plateau, F. femur condyle, ST, synovial tissue. **A**, **B and C** images were taken using 1.25x microscope lenses.

We performed immunostaining using IL-6 and CCL21-antibodies to determine the effect of MMD on the protein expression of the two cytokines in rat knees collected at 3 days post-surgery. While IL-6 was detected mostly at synovial tissue in the medial side of MMD-operated knees, it was nearly undetectable at the collateral side and in the control sham-operated knee joints (Fig 5), CCL21 was detected in synovial tissue as well as articular cartilage in MMD-knees (Fig 6), and it was mostly found around vascularization in synovial tissue of control sham-operated knees. Very weak expression of CCL21 was detected in articular cartilage of control sham-operated knees (Fig 6).

Since qPCR data showed significant increase in *Ccl21* mRNA level both at early and later stages during PTOA development compared to control un-operated knees, we performed immunostaining using CCL21-antibody on histology sections prepared from rat knee joints collected at 4 week-post MMD, CCL21 was highly expressed in synovial tissue, around vascular tissue and in articular cartilage of MMD-knee joints (Fig 6) compared to control knees.

### Effect of CCL21 blockade on articular cartilage degeneration

**Fig 7:**
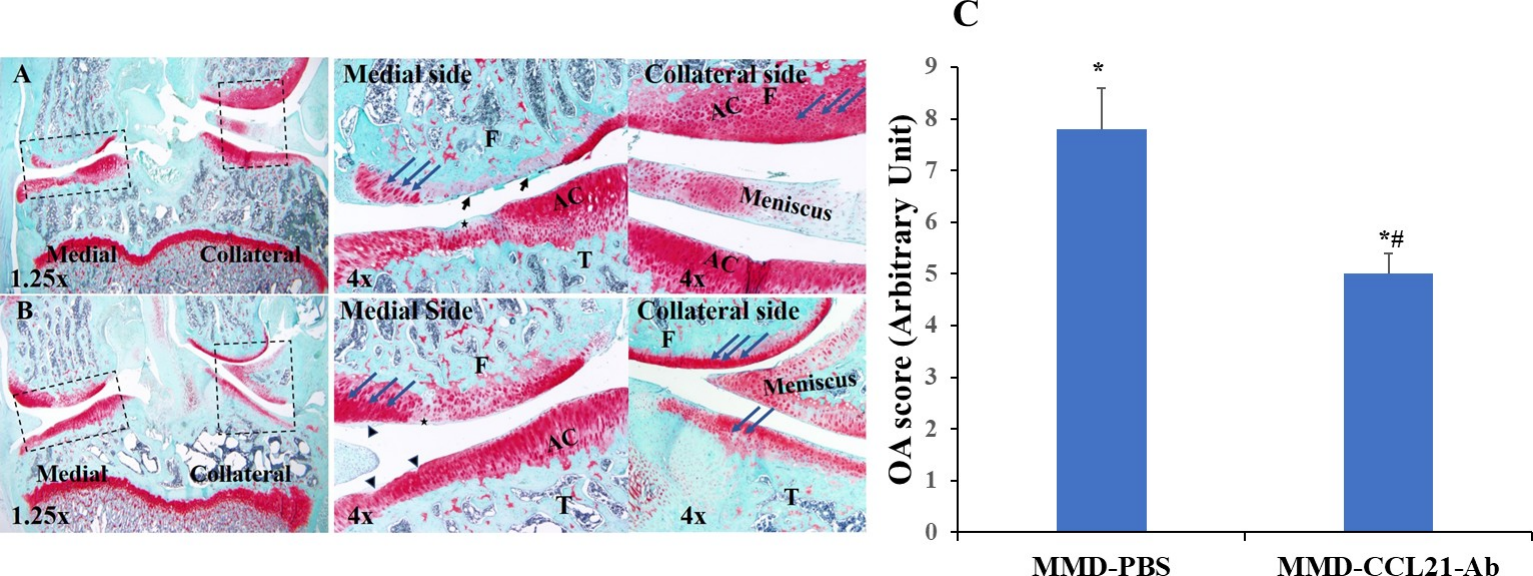
Treatment with CCL21 antibody ameliorates MMD-induced PTOA development in rat knees. Representative images from histology sections isolated, at 4-wks post-surgery from MMD knees treated with PBS (**A**) showing irregularities, fibrillations at the articular cartilage (black arrows) and loss of safranin-o staining (black stars) at the medial side. The MMD-knees treated with10 μg CCL21-Ab (**B**), showing fewer irregularities and thinner fibrillations (black arrowheads) at the superficial zone of the articular cartilage and less loss of safranin-O staining (black stars). Bleu arrows show tide mark line. Sections were stained with Safranin-O/fast green and observed using microscope objectives 1.25x (**A** and **B**) and 4x (**right panels**). T. tibia plateau and F. femur condyle. AC for articular cartilage. Cartilage damage quantification (**C**). Histopathology Evaluation of MMD-operated knees at Four Week- Post-Surgery, for PBS and CCL21-Ab treated rats. Data are expressed as mean ± SE, n = 6–8, *P<0.05 vs controls, #P<0.05 vs MMD-PBS.

To determine if CCL21 plays a role in PTOA development, we have used MMD rat model and CCL21 antibodies. The right knee of each rat was MMD-operated, and the left knee was un-operated and used as control. One group was treated with CCL21-antibody (Ab) and the other group was treated with PBS and was used as MMD-control. Injections of PBS or CCL21-Ab were performed under the patella, at the anteromedial knee between the condyle and the tibia plate, and towards the medial side of the knee. Animals received one injection per week for four weeks, starting from day one post-surgery to block the function of CCL21 early post-surgery. Histology assessment of knee joints at 4 week-post surgery (Fig 7A), showed irregularities, fibrillations at the articular cartilage and proteoglycan loss as evidenced by loss of safranin-o staining obvious at the medial side in MMD knees treated with PBS. Less proteoglycan loss and cartilage degradation in response to MMD was observed in the knees treated with CCL21-Ab (Fig 7B and 7C) as compared to MMD- PBS treated knees. Fewer irregularities were observed at the superficial zone of the articular cartilage of the MMD-knees treated with CCL21-Ab (Fig 7B), compared to control MMD knees, and OA scores were significantly reduced in MMD-knees treated with CCL21-Ab compared to MMD-knees treated with PBS (Fig 7C).

### Effect of CCL21 blockade on inflammation and cartilage degrading enzyme expression post-MMD

**Fig 8:**
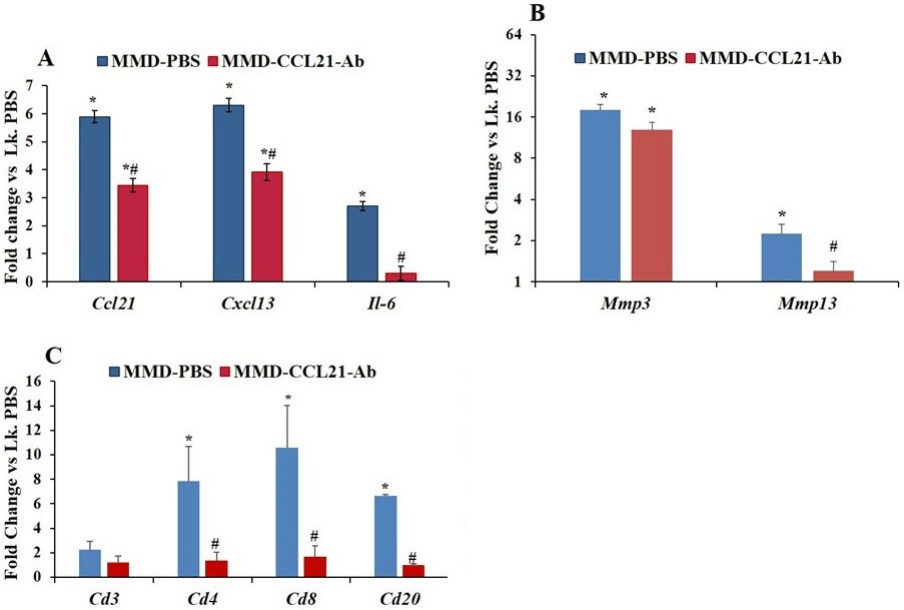
The effect of local blockade of CCL21 function, on inflammation in rat knees, at three days post-surgery. mRNA levels of the major pro-inflammatory cytokine *Il-6* and the chemokines *Ccl21* and *Cxcl13* **(A)**, *Mmp3* and *Mmp13* **(B)** and the markers of four inflammatory cell subsets **(C).** Right knees (Rk) were MMD-operated and left knees (Lk) were control un-operated from PBS treated rats. Data are expressed as fold change of the expression of each gene in the Rk treated with either 10 μg CCL21-Ab or PBS *vs* Lk of rats treated with PBS (Lk. PBS) and are represented as mean ± SE. n = 3–5, *P≤0.05 *vs* Lk. PBS and #P≤0.05 Rk-CCL21-Ab *vs* Rk-PBS.

To determine if early inflammation is important for PTOA development and if CCL21 is responsible for the recruitment of inflammatory cells and subsequent PTOA development, we used a CCL21-Ab to block CCL21 function, and then evaluated the changes in inflammation and expression levels of cartilage degrading enzymes at different time points. Antibodies were administered at one day post-surgery to reduce inflammatory cell infiltration to the operated knee joints in response to injury and compared against PBS control. The results of qPCR showed that while mRNA levels of *Ccl21*, *Cxcl13* (Fig 8A), and *Mmp3* (Fig 8B**)**, were significantly increased in both CCL21-Ab and PBS treated MMD-knees as compared to unoperated knees at 3- days post-surgery, mRNA levels of *Il-6* and *Mmp13* were increased in MMD-knees treated with PBS but not CCL21-Ab treated MMD-knees compared to control unoperated knees (Fig 8) at 3-days post-surgery (Fig 8A and 8B).

We have also evaluated the mRNA levels of three T cell markers and one B lymphocyte marker in whole knees, including synovial tissue, articular cartilage and epiphyseal bone. RNA was isolated from MMD operated knees and control unoperated knees at day 3 post-surgery. 8-10-fold increase in mRNA level of *Cd4* (P = 0.02) and *Cd8* (P = 0.048) was found, in MMD-knees treated with PBS compared to control unoperated knees (Fig 8C). mRNA level of *Cd20* was 6-fold increased in MMD-operated knees treated with PBS compared to control un-operated knees (Fig 8C; P = 0.034), but no significant difference was detected in mRNA levels of *Cd3* between MMD- and control un-operated knees at 3 days post-surgery. Significantly, mRNA levels of *Cd4*, *Cd8* and *Cd20* (Fig 8C) were 5-fold down-regulated in MMD-knees treated with CCL21-Ab compared to control MMD-knees. These data suggest that blocking the function of CCL21 inhibits chemotaxis function of CCL21 and in turns reduces infiltration of both CD4+, CD8+ and B+ cells to the site of injury. This ultimately leads to a reduction in *Mmp13* mRNA expression (Fig 8B) at day 3 post-surgery.

**Fig 9:**
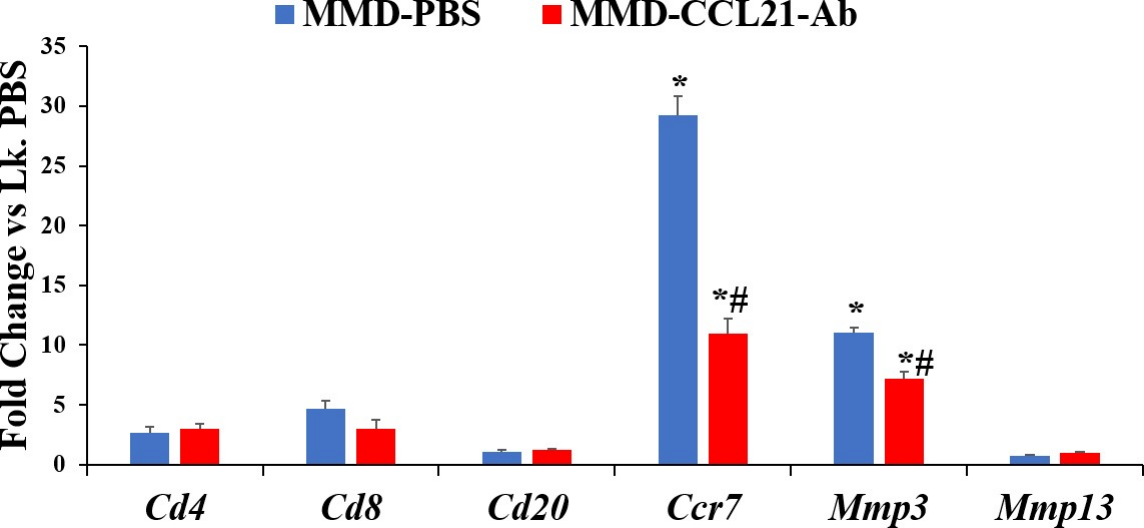
Effect of blocking the function of CCL21 in rat knees, on *Mmp3* and *Mmp13* and four markers of inflammatory cell subsets, at day five post-knee surgery. Right knees (Rk) were MMD-operated and left knees (Lk) were un-operated. Data are expressed as fold change of the expression of each gene in the Rk treated with either CCL21-Ab or PBS *vs* Lk. PBS and are represented as mean ± SE. n = 4, *P≤0.05 *vs* Lk. PBS and #P≤0.05 Rk-CCL21-Ab *vs* Rk-PBS.

At day-five- post-surgery (Fig 9), mRNA levels of *Ccr7* and *Mmp3* were significantly increased in both MMD-PBS and MMD-CCL21-Ab treated knees compared to control un-operated knees, but this change in expression was significantly greater in MMD-PBS treated knees compared to MMD-CCL21-Ab treated knees for both *Ccr7* and *Mmp3* genes. Surprisingly, no significant change was observed in mRNA levels of *Cd4* and *Cd8* between MMD and control unoperated knees for both PBS and CCL21-Ab treated MMD-knees at this time point (Fig 9).

### CD4 and CD8 infiltration in response to MMD and CCL21-Ab treatment-immunohistochemical staining

**Fig 10:**
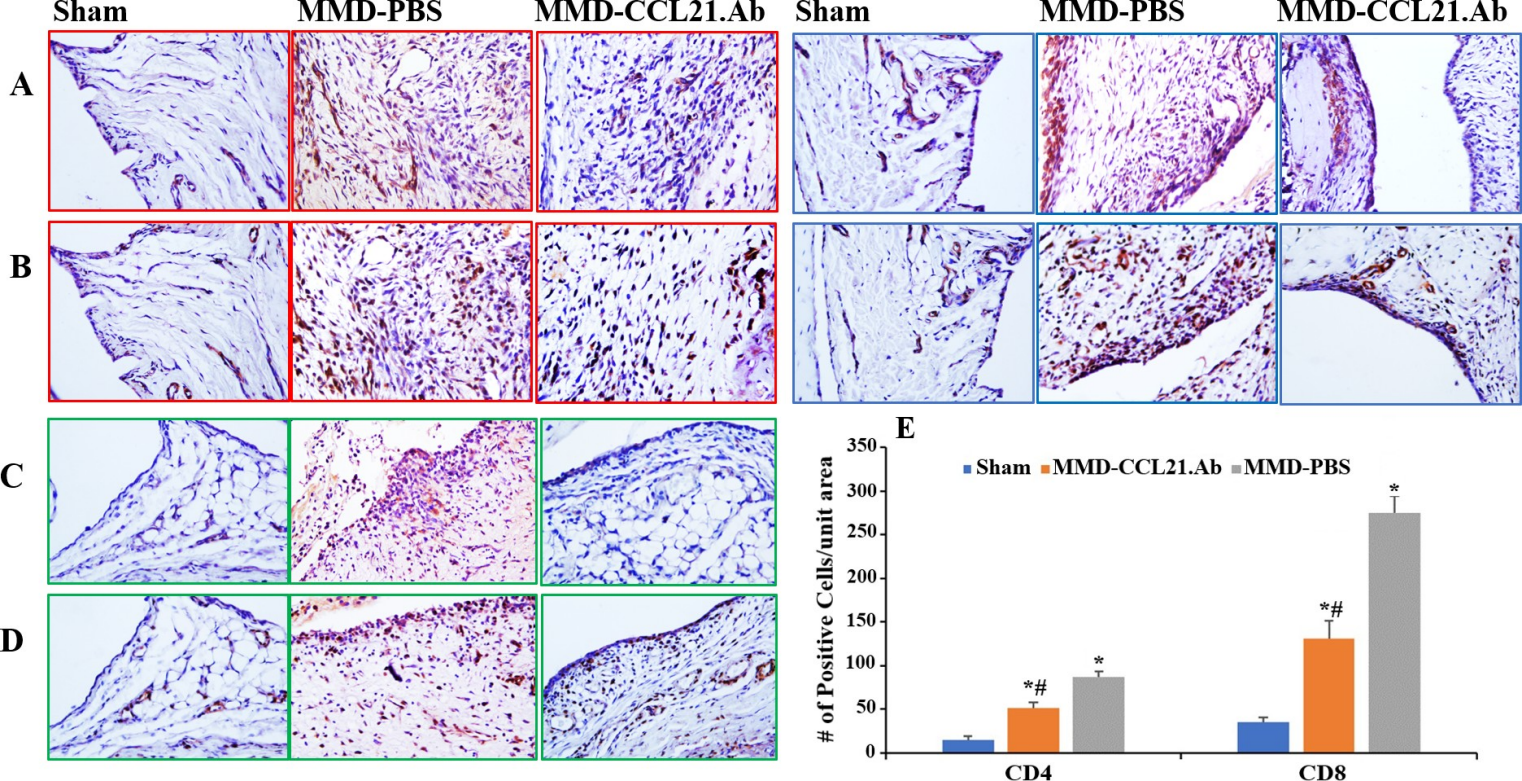
MMD induced T cell infiltration to rat injured knees and treatment with CCL21-Ab reduced the number of CD4+ and CD8+ cells in MMD-knees at day 3 post surgery. Images of areas from knee joints’ synovial tissues collected from; Sham-, control MMD-knees treated with PBS (MMD-PBS). and MMD-knees treated with CCL21-Ab (MMD-CCL21.Ab). Histology sections collected from rat knees at day 3 post-surgery were used. Immuno-histochemistry staining was performed using rat CD4 (A and C) and CD8 (B and D) antibodies, and counterstained with hematoxylin. Images in red squares represent areas from medial side of knee joints. Images in blue squares represent areas from collateral side of the knee joints. Images in green squares were taken at the frontal end of joint capsule, near the connection of the Patella ligament with femur. The exact position in knee joints is indicated in S1 Fig. The graph in the right bottom panel represents the number of positive stained cells per unit area at the joint capsules (E). Data are expressed as number of positive stained cells per unit area and are presented as mean ± SE. *P<0.05 vs Lk sham-operated knees. #P<0.05 vs MMD-saline, n = 5–6 knees.

qPCR data showed significant increase in *Cd4* and *Cd8* mRNA levels post-MMD (Fig 8C). Treatment with CCL21-Ab reduced the levels of both *Cd4* and *Cd8* mRNA in MMD-knees compared to control PBS treated MMD-knees early post-surgery (Fig 8C). To determine if this effect on the mRNA levels is due to CCL21-Ab-induced reduction in T cell recruitment, we performed immunohistochemistry staining with antibodies against the two T cell subsets; CD4 and CD8 using histologic sections collected from sham-operated knees and both CCL21-Ab and PBS treated MMD-knees (Fig 10 and S1 Fig). Immunohistochemistry staining using CD4 and CD8 antibodies showed a significant increase in CD4+ and CD8+ cells in the joint capsule of both CCL21-Ab and control MMD-knees compared to control sham-operated knees (Fig 10), no positive staining was observed in normal goat serum treated sections that was used as a negative control (S2 Fig). CD4+ and CD8+ cells were found to be much more abundant in MMD-knee joints compared to sham-operated knee joints (Fig 10). The density of positive stained cells was quantified at both medial and collateral sides of the knee joints as well as the frontal side of the joint capsule (Fig 10). The number of CD4+ and CD8+ cells per unit area was significantly increased in both MMD-knee groups compared to control sham-operated knees, at day 3 post-surgery (Fig 10E). However, the magnitude of increase was significantly reduced in CCL21-Ab treated MMD knees compared to PBS-MMD knees (Fig 10E). The difference in the density of positive cells between Ab treated and control treated MMD-knees was more obvious at the frontal side of the joint capsules (Fig 10C and 10D).

## Discussion

Traumatic meniscal injury induces a chronic inflammatory response in injured knees [14–16]. In our experimental model, altering the macrostructure of the meniscus by removing piece of the meniscus and cutting the connection meniscus-tibio-femoral causing meniscal and knee destabilization affects the ability of the menisci to constrain the knee to “normal” motion during daily activities. This led to excessive loading and contribute to the pathogenesis of PTOA. While it is now accepted that early inflammation post knee injury plays an important role in the development of PTOA [17], the molecular pathways involved are still not well understood, so this study’s focus was to analyze the role of CCL21 on post-MMD inflammation and development of PTOA.

It has been reported that knee joints that developed PTOA post MMD showed increased expression of *Ccl21* compared to healthy knees [3–5]. CCL21 with CCL19 are the only chemokine ligands for chemokine receptor 7 (CCR7) [7] that is expressed in CD4+ T cell subset and dendritic cells. For the first time, we report here, the time course expression pattern of *Ccl21*, from day 1- to eight week-post-MMD and during PTOA development, in rat and mouse MMD models. Our results showed that *Ccl21* mRNA expression was elevated by MMD-induced injury and remains greater than healthy knees for at least 8 weeks. These results suggest a potential role for CCL21 in not only MMD-induced inflammation but also in the subsequent cartilage degeneration and osteophyte formation processes.

To test for the cause-and-effect relationship between changes in *Ccl21* expression, and inflammation and articular cartilage degradation, we evaluated the consequences of blockade of CCL21 function using a neutralizing antibody. We chose local injection of antibodies for several reasons. First, local delivery increases bioavailability at the site of injection by minimizing the exposure of the antibodies to enzymatic degradation in the blood circulation. Second, local delivery increases efficacy by reducing the loss of the antibody due to non-specific binding in non-target tissues. Third, local delivery increases specificity by minimizing off-target effects of the antibody in other tissues. Moreover, intra-articular injection of test substances has been routinely used in OA studies for rodents and humans [18, 19].

The results of our study showed that treatment of MMD knees with CCL21 antibodies altered mRNA expression of *Mmps* and mitigated post-MMD inflammation as evidenced by a reduction in the expression of *Il-6* and a reduction in inflammatory cell recruitment to the joint of MMD operated knees. These data together suggest that expression of the major cartilage degrading enzymes *Mmp3* and *Mmp13* is dependent on *Ccl21* expression. This is likely since CCL21 has a chemotactic effect on inflammatory cells that secret MMPs and thus, alteration of CCL21function will limit cell infiltration and MMPs secretion into the injury site.

A strong cooperation between T helper cells that express CD4 in their membrane and the B cells known to express CD20 has been previously reported [20]. Moreover, studies with OA patients showed notable fractions of CD20+ cells in synovial tissue of OA patients, and increased levels of CD20 cells in the subchondral marrow tissues of OA knees compared to healthy knees [21]. Based on our findings that the expression levels of *Cd4* and *Cd8* markers were altered in the MMD-knees treated with CCL21-Ab, we tested if inhibition of CCL21 action also affected recruitment of CD20+ cells by measuring *Cd20* mRNA level in the knee joints. We found that neutralizing the function of CCL21 caused a reduction in the expression of *Cd20* mRNA levels in the MMD-operated knees compared to MMD-knees treated with PBS, at three days post-surgery. At day five post-surgery, when the mRNA level of *Cd4* went down similarly in both groups, *Cd20* mRNA level was also reduced compared to its level at three days post-surgery, confirming the cooperation between CD4+ andCD20+ cells. In addition, at day five-post-surgery, while the mRNA level of *Mmp3* and *Ccr7* was still greater in both MMD-knee groups compared to control un-operated knees, it was reduced in CCL21-Ab treated MMD-knees compared PBS treated MMD-knees, confirming the relationship between T cells and matrix degradation. Furthermore, CCR7 is expressed by T cells and was also found in synovial fibroblasts [22]. In response to its ligands CCL19 or CCL21, CCR7 mediates upregulation of vascular endothelial growth factor (VEGF) suggesting a role in synovial angiogenesis in arthritic knees [22].

Previous studies showed that lack of *Ccr7* (receptor for CCL21) expression in *Ccr7*-KO mice resulted in protection from early cartilage degeneration and from osteophyte formation at 6 weeks post-MMD compared to control wild type mice [23]. In the present study, we have shown for the first time in an MMD rat model that knee joint treatment with neutralizing antibody against the CCR7 ligand, CCL21, is effective to significantly reduce early inflammation in response to knee injury. Furthermore, CCL21-Ab treatment significantly reduced OA score as determined by histopathological evaluation of the injured knees. However, in the present study, we did not test the long-term effect of CCL21-Ab on PTOA development and osteophyte formation, and most of our work was based on gene expression level immediately after injury, and on histology phenotype. The consequence of neutralization of CCL21 action on immune cell sub types was not quantitated in the synovium during PTOA development. Furthermore, CCL21-Ab effect on pain was not investigated in this study. Thus, more investigations are needed to confirm the extent to which neutralization of CCL21 action provides therapeutic effect on PTOA development and on the mechanisms by which CCL21-Ab confers its protective effect on PTOA development.

Recent studies by Joutoku et al. [24], using a different animal model showed that local administration of CCL21 to adult rabbits at the knee joints can enhance cartilage repair post osteochondral defect. In Joutoku et al. [24] studies, the experimental defect affected both articular cartilage and osteochondral bone and was deep enough to reach bone marrow [24]. This allowed the infiltration of mesenchymal stem cells from bone marrow to the injured knee joints for cartilage regeneration. In our experimental model, this is not possible as MMD-induced injury affects only the medial meniscus. Together, these data show the importance of early post-knee injury inflammation on PTOA development and reveal the complexity of CCL21 function that can influence the recruitment of both inflammatory cells from blood circulation and mesenchymal stem cells from bone marrow to the injured knee joint. Understanding the molecular pathways for CCL21-CCR7 action in regulating the migration of different cell types may reveal new avenues of inquiry in the development of new therapeutic approaches effective to prevent and treat PTOA.

## Supporting information

S1 Table Time post-surgery of different assays and animal species used for each assay and at each time point. (DOCX)

S2 Table Sequences of the primers used in this study. (DOCX)

S1 Fig Images of histology sections from immunohistochemistry staining using CD4 and CD8 antibodies. Histology sections were collected, at day 3 post-surgery, from sham-, MMD-knees treated with PBS (MMD-PBS) and MMD-knees treated with CCL21-ab (MMD-CCL21.Ab). Immunohistochemistry staining was performed using rabbit anti-rat CD4 and mouse anti-rat CD8 primary antibodies. Brown colored areas show positive stained areas. Left panels represent images of knee joint sections taken using 1.25x microscope lenses and images in red and blue squares represent the areas magnified 20x on the right panels; medial and collateral. The images in green squares represent the areas indicated on the left at the frontal side of the joint capsule. F. femur condyle; black arrows show some positive stained areas.
(DOCX)

S2 Fig Images of histology sections from immunohistochemistry staining using normal goat serum as negative control. Histology sections were collected, at day 3 post-surgery, from sham- (**A** and **D**), MMD-knees treated with PBS (**B** and **E**) and MMD-knees treated with CCL21-ab (**C** and **F**). Immunohistochemistry staining was performed using primary antibodies and normal goat serum, only images from serum treated sections are presented in this figure. Left panels represent images taken using 1.25x microscope lenses, red and blue squares represent the areas magnified 20x on the right panels; medial and collateral. The images in green squares represent the magnified areas indicated on the left at the frontal side of the joint capsule. F. femur condyle; ST. synovial tissue.
(DOCX)
