# Correction: Evaluation of CCL21 role in post-knee injury inflammation and early cartilage degeneration

**DOI:** 10.1371/journal.pone.0259553

**Published:** 2021-10-28

**Authors:** Subburaman Mohan, Bouchra Edderkaoui

The first author’s name is listed incorrectly. Their correct name is: Subburaman Mohan.

The correct citation is: Mohan S, Edderkaoui B (2021) Evaluation of CCL21 role in post-knee injury inflammation and early cartilage degeneration. PLoS ONE 16(3): e0247913. https://doi.org/10.1371/journal.pone.0247913
