# Supplementary material for: Evaluation of CCL21 role in post-knee injury inflammation and early cartilage degeneration
Source: PLoS One. 2021 Mar 2;16(3):e0247913. doi: 10.1371/journal.pone.0247913 (PMC7924772; doi:10.1371/journal.pone.0247913)
Supplement: S1 Table — (DOCX) [file pone.0247913.s001.docx]

**S1 Table.** **Time post-surgery of different assays and animal species used for each assay and at each time point.**

| **Assay-species-Tissue/Time points(days/wks post surgery)** | **1 day** | **3 days** | **5 days** | **1 wk** | **2 wks** | **4 wks** | **6 wks** | **8 wks** |
| --- | --- | --- | --- | --- | --- | --- | --- | --- |
| qPCR-rats from whole knees | x | x | x |  |  | x |  |  |
| qPCR-rats synovial tissue from MMD and sham-knees | x |  |  |  |  |  |  |  |
| qPCR-mice from whole knees -Ccl21 only |  |  |  | x | x | x |  | x |
| Histology-rat knee joints | x | x |  |  |  | x | x |  |
| Immunostaining for rat knee joints |  | x |  |  |  | x |  |  |

wks for weeks post-surgery.
