# Supplementary material for: Evaluation of CCL21 role in post-knee injury inflammation and early cartilage degeneration
Source: PLoS One. 2021 Mar 2;16(3):e0247913. doi: 10.1371/journal.pone.0247913 (PMC7924772; doi:10.1371/journal.pone.0247913)
Supplement: S2 Table — (DOCX) [file pone.0247913.s002.docx]

**S2 Table. Sequences of the primers used in this study**

| **Primer** | Forward | Reverse |
| --- | --- | --- |
| *Ccl21* | CAT GGC CGT CCC TTT CTT CT | TCT TGG GAC CTG AGT GAC CT |
| *Ccr7* | GCT GGT GGT GCT GAC ATA CA | CAG GAC TTG GCT TCG CTG TA |
| *Cd3* | CTG GTG CTA GAG GAT TTC TC | ATG GAT ACT GCT GTC AGG TC |
| *Cd4* | AGG GAG AGT TGA GAT GGA AG | TAT CTG AAG GGT GAG TGG GA |
| *Cd8* | GAG CAA GCT GAA CGA TAT A | TTC ACT TTC TGA AAC ACC G |
| *Cd20* | TAAGCCTCTTTGCTGCCATT | GGAATTGGATGGCTCACAGT |
| *Cxcl13* | CTC CAG GCC ACG GTA TTC TG | CAG TTT TGG GGC AGC CAT TC |
| *IL-6* | TAC CCC AAC TTC CAA TGC TC | GGT TTG CCG AGT AGA CCT CA |
| *Mmp13* | TGC GGT TCA CTT TGA GGA CA | TTT GTC GCC AAT TCC AGG GA |
| *Mmp3* | CTG CGG GGA GAA GTC TTG TT | TGT TGG ATG GAA GAG ACG GC |
| *Ppia* | TCT GCA CTG CCA AGA CTG AG | ATT CCT GGA CCC AAA ACG CT |
| *Tnf-a* | TGC CTC AGC CTC TTC TCA TT | CCC ATT TGG GAA CTT CTC CT |
| *Ppia*-mouse | CCATGGCAAATGCTGGACCA | TCCTGGACCCAAAACGCTCC |
| *Ccl21*-mouse | TCCCGGCAATCCTGTTCTC | TTCTGCACCCAGCCTTCCT |

All primers are from Rattus norvegicus except when it’s stated mouse
