# Supplementary material for: Evaluation of CCL21 role in post-knee injury inflammation and early cartilage degeneration
Source: PLoS One. 2021 Mar 2;16(3):e0247913. doi: 10.1371/journal.pone.0247913 (PMC7924772; doi:10.1371/journal.pone.0247913)
Supplement: S1 Fig — Histology sections were collected, at day 3 post-surgery, from sham-, MMD-knees treated with PBS (MMD-PBS) and MMD-knees treated with CCL21-ab (MMD-CCL21.Ab). Immunohistochemistry staining was performed using rabbit anti-rat CD4 and mouse anti-rat CD8 primary antibodies. Brown colored areas show positive stained areas. Left panels represent images of knee joint sections taken using 1.25x microscope lenses and images in red and blue squares represent the areas magnified 20x on the right panels; medial and collateral. The images in green squares represent the areas indicated on the left at the frontal side of the joint capsule. F. femur condyle; black arrows show some positive stained areas. (DOCX) [file pone.0247913.s003.docx]

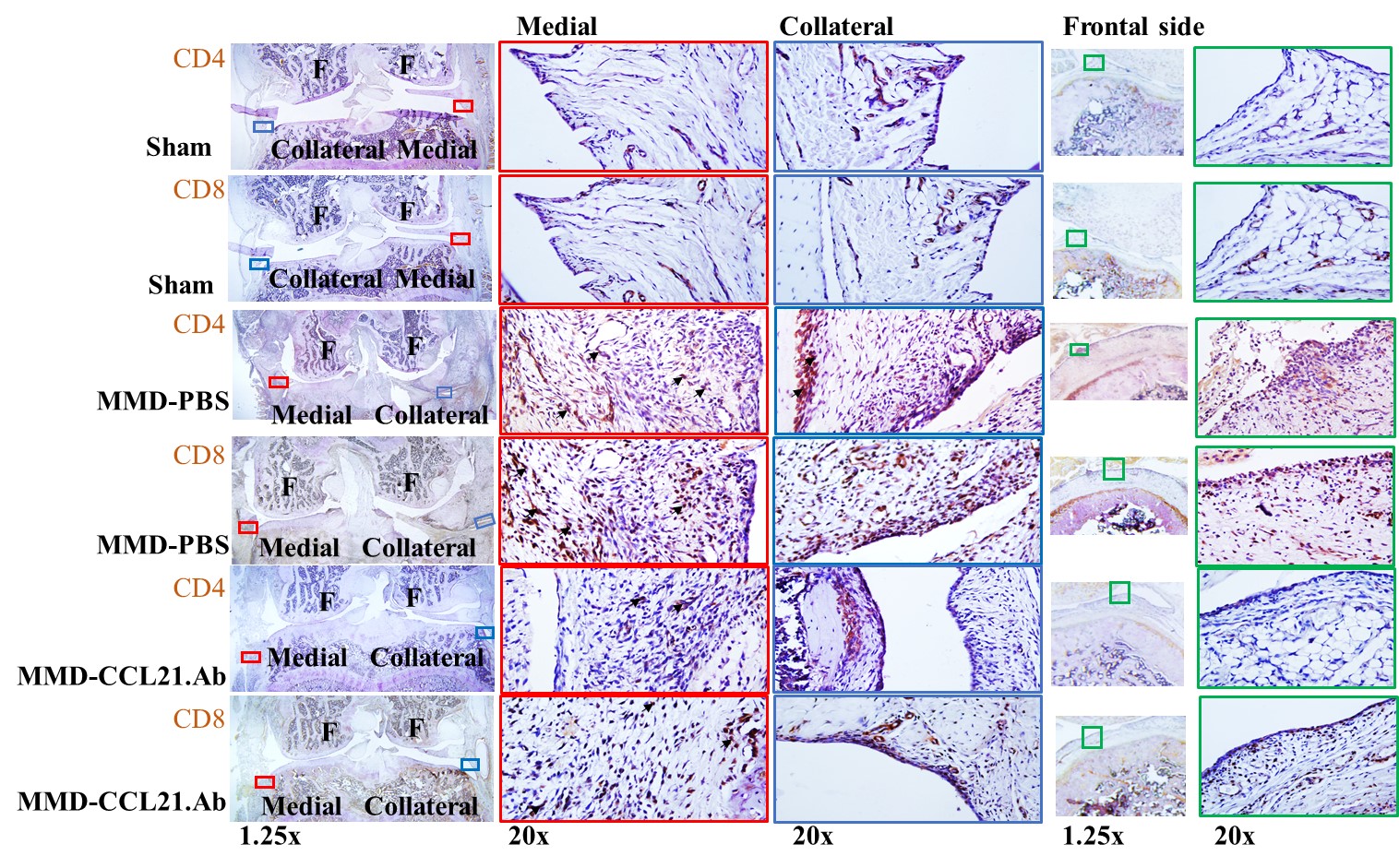


**S1 Fig.** **Images of histology sections from immunohistochemistry staining using CD4 and CD8 antibodies**. Histology sections were collected, at day 3 post-surgery, from sham-, MMD-knees treated with PBS (MMD-PBS) and MMD-knees treated with CCL21-ab (MMD-CCL21.Ab). Immunohistochemistry staining was performed using rabbit anti-rat CD4 and mouse anti-rat CD8 primary antibodies. Brown colored areas show positive stained areas. Left panels represent images of knee joint sections taken using 1.25x microscope lenses and images in red and blue squares represent the areas magnified 20x on the right panels; medial and collateral. The images in green squares represent the areas indicated on the left at the frontal side of the joint capsule. F. femur condyle; black arrows show some positive stained areas.
