# Supplementary material for: Evaluation of CCL21 role in post-knee injury inflammation and early cartilage degeneration
Source: PLoS One. 2021 Mar 2;16(3):e0247913. doi: 10.1371/journal.pone.0247913 (PMC7924772; doi:10.1371/journal.pone.0247913)
Supplement: S2 Fig — Histology sections were collected, at day 3 post-surgery, from sham- (A and D), MMD-knees treated with PBS (B and E) and MMD-knees treated with CCL21-ab (C and F). Immunohistochemistry staining was performed using primary antibodies and normal goat serum, only images from serum treated sections are presented in this figure. Left panels represent images taken using 1.25x microscope lenses, red and blue squares represent the areas magnified 20x on the right panels; medial and collateral. The images in green squares represent the magnified areas indicated on the left at the frontal side of the joint capsule. F. femur condyle; ST. synovial tissue. (DOCX) [file pone.0247913.s004.docx]

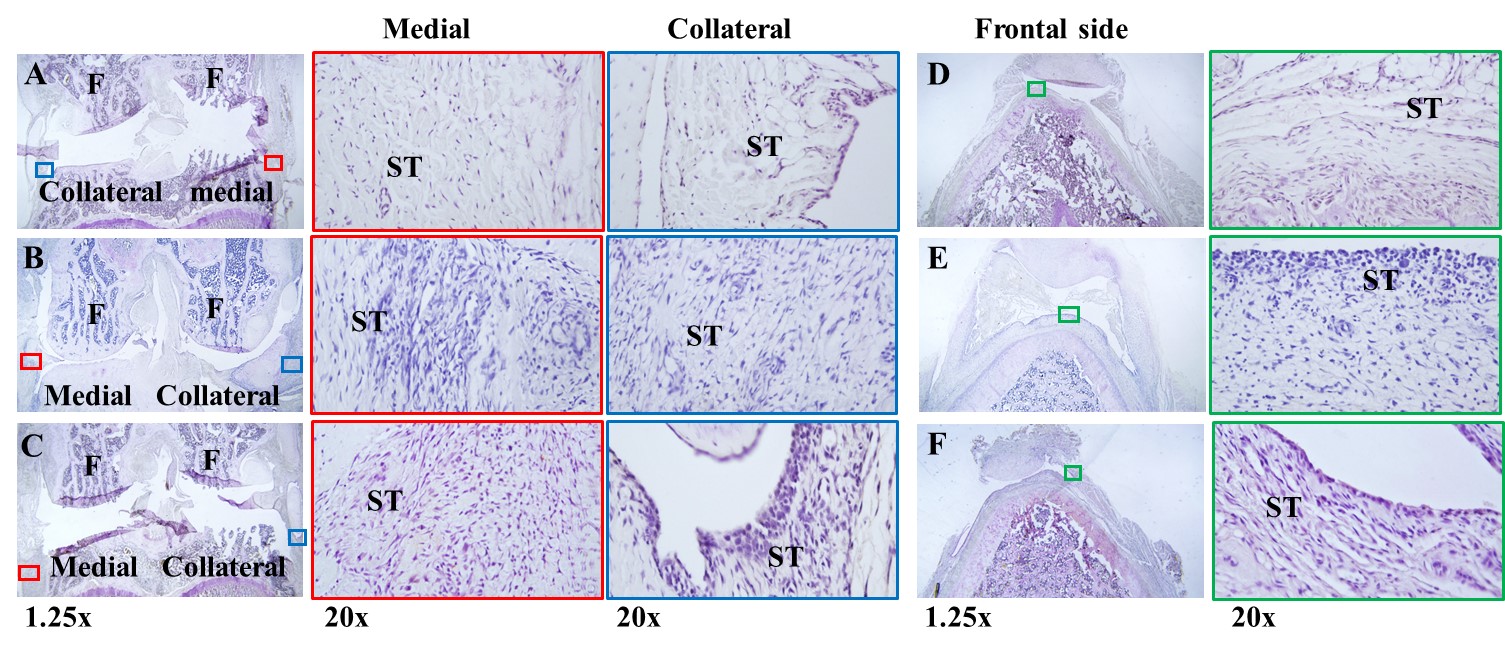


**S2 Fig.**  **Images of histology sections from immunohistochemistry staining using normal goat serum as negative control.** Histology sections were collected, at day 3 post-surgery, from sham- (**A** and **D**), MMD-knees treated with PBS (**B** and **E**) and MMD-knees treated with CCL21-ab (**C** and **F**). Immunohistochemistry staining was performed using primary antibodies and normal goat serum, only images from serum treated sections are presented in this figure. Left panels represent images taken using 1.25x microscope lenses, red and blue squares represent the areas magnified 20x on the right panels; medial and collateral. The images in green squares represent the magnified areas indicated on the left at the frontal side of the joint capsule. F. femur condyle; ST. synovial tissue.
